# Feature-Based Normality Models for Anomaly Detection

**DOI:** 10.3390/s25154757

**Published:** 2025-08-01

**Authors:** Hui Yie Teh, Kevin I-Kai Wang, Andreas W. Kempa-Liehr

**Affiliations:** 1 Department of Electrical, Computer and Software Engineering, The University of Auckland, Auckland 1142, New Zealandkevin.wang@auckland.ac.nz (K.I.-K.W.); 2 Department of Engineering Science and Biomedical Engineering, The University of Auckland, Auckland 1142, New Zealand

**Keywords:** anomaly detection, feature engineering, normality model, sensor data quality, time series analytics

## Abstract

Detecting previously unseen anomalies in sensor data is a challenging problem for artificial intelligence when sensor-specific and deployment-specific characteristics of the time series need to be learned from a short calibration period. From the application point of view, this challenge becomes increasingly important because many applications are gravitating towards utilising low-cost sensors for Internet of Things deployments. While these sensors offer cost-effectiveness and customisation, their data quality does not match that of their high-end counterparts. To improve sensor data quality while addressing the challenges of anomaly detection in Internet of Things applications, we present an anomaly detection framework that learns a normality model of sensor data. The framework models the typical behaviour of individual sensors, which is crucial for the reliable detection of sensor data anomalies, especially when dealing with sensors observing significantly different signal characteristics. Our framework learns sensor-specific normality models from a small set of anomaly-free training data while employing an unsupervised feature engineering approach to select statistically significant features. The selected features are subsequently used to train a Local Outlier Factor anomaly detection model, which adaptively determines the boundary separating normal data from anomalies. The proposed anomaly detection framework is evaluated on three real-world public environmental monitoring datasets with heterogeneous sensor readings. The sensor-specific normality models are learned from extremely short calibration periods (as short as the first 3 days or 10% of the total recorded data) and outperform four other state-of-the-art anomaly detection approaches with respect to F1-score (between 5.4% and 9.3% better) and Matthews correlation coefficient (between 4.0% and 7.6% better).

## 1. Introduction

With the ubiquity of Internet of Things (IoT) applications, the need for good sensor data quality has become increasingly critical to ensure the success of these data-driven applications. Typical IoT applications, such as environmental and structural health monitoring [1,2,3], healthcare [4,5], and smart cities [6,7], generate vast amounts of data from the hundreds or thousands of sensor devices in wireless sensor networks (WSNs). An individual sensor device may often contain several sensors that measure physical properties such as temperature, motion, pressure, infrared radiation, and chemicals [8] at a predetermined sampling frequency.

Increasingly, there is a shift to deploy low-cost sensors in some fields of IoT, such as environmental monitoring, as opposed to the more costly high-end industrial sensors [9,10,11,12]. The key attractiveness of low-cost sensors is the customisation and personalisation benefits, which allow end-users to fully tailor the network application based on their needs, on top of the obvious cost benefits. However, they are not as robust as their high-end counterparts and are more likely to exhibit faster degradation [13,14].

Therefore, ensuring sensor data quality for IoT applications utilising low-cost sensors is vital. Without good data quality, the erroneous data collected may burden the already overwhelmed network infrastructures and central storage space. Poor sensor data quality also affects machine learning models, dramatically impacting the decision-making results of IoT applications. Anomaly detection, which distinguishes between normal and anomalous data, allows us to weed out irregular data automatically, making data collection more efficient and cost-effective by improving sensor data quality. It also supports proactive measures, such as sending an alert or performing imputation to maintain the reliability and integrity of sensor readings.

Automated anomaly detection in IoT sensor data poses three major difficulties:

1. Unexpected types of anomalies can occur.
2. Every deployed sensor will exhibit expected but potentially unknown application-specific and location-specific deviations from signals generated under laboratory conditions.
3. Only short calibration periods of sensor data are available in real-world applications, assuming that an engineer can set up and monitor the sensors in a short deployment period a few days after installation.

Recent solutions [1,6,15,16,17,18,19] proposed to solve the anomaly detection problems are partially automated and are not adaptive to deployed and distributed sensors. The inputs used for the machine learning models in the proposed anomaly detection solutions either are manually engineered features requiring domain knowledge from experts, which can be hard to obtain, or require a large amount of training data. The manually engineered features may also not be generalisable if the sensors are deployed in heterogeneous environmental conditions. Though Harandi et al. [20] tackle this issue by using automatic feature extraction via signal processing features, they use supervised methods requiring labelled datasets, which are difficult to obtain and are likely to be ignorant of unknown anomaly types.

Due to varying sensor behaviours, learning the normal behaviour of a sensor can be challenging for anomaly detection methods such as Principal Component Analysis (PCA) [21]. In addition, most state-of-the-art approaches that involve clustering [6,17,22,23,24,25] require the data to be sent to the cloud or server side to have the anomaly detection performed retrospectively. They are not processed in real time on sensing or edge devices. Lastly, some supervised methods [20,26,27,28,29] require lots of training data with labelled anomalies, which are difficult to obtain in practice.

To address these challenges and allow adaptive and fully automated anomaly detection, our study combines systematic time series feature engineering with unsupervised feature selection to learn sensor-specific normality models from short calibration periods used as training data. The proposed method, *Adaptive and Automated Anomaly Detection* (AAAD), combines the unsupervised learning approach presented in [30] with one-class Local Outlier Factor (LOF) [31] classifiers. During a short calibration period (as short as the first 3 days or 10% of the total recorded data), the proposed learning framework trains on anomaly-free data, where the normal behaviour of a sensor time series is learnt. The calibrated model can then be deployed to detect unprecedented anomalies that the model has never seen before.

In summary, the contribution of our paper is three-fold:

- A sensor-specific anomaly detection framework that learns a normality model of the sensor dynamics, allowing anomaly detection to be adaptive for distributed sensor devices;
- A comprehensive evaluation of our anomaly detection model compared to other state-of-the-art anomaly detection models, including one-class classifiers and clustering approaches over six evaluation metrics;
- The demonstration of the applicability and robustness of the developed framework on different types of complex anomalies, such as point, contextual, and collective anomalies, on three public environmental monitoring datasets.

The rest of this paper is organised as follows. Section 2 discusses the recent state-of-the-art anomaly detection methods involving one-class classifiers. Section 3 details our proposed adaptive anomaly detection framework. Section 4 describes the experimental setup and the publicly available datasets used for evaluation, which involve all three types of anomalies, and the results of the experiments are shown and discussed in Section 5. Section 6 concludes this paper.

## 2. Related Works

There has been extensive interest in and work performed on anomaly detection in sensor-data-quality-related problems [32,33,34,35,36], which is put into the context of our contribution in this section. A robust unsupervised feature engineering approach ws developed [30], which extracts an extensive set of statistically relevant time series features. The features are selected in the feature selection step, which does not require ground-truth labels for anomaly detection problems. Though the features are automatically selected without domain knowledge and are generalisable across sensor time series data, the anomaly detection model has to be able to work with varying sensor behaviours.

Regarding anomaly detection techniques, one-class classifiers [37] have become popular for anomaly detection in IoT sensor data; these classifiers can identify a non-linear boundary separating normal and anomalous data. One-class classifiers implement a special case of classification where the training samples come from a single positive class, hence the name “one-class”. In sensor data anomaly detection, the input for one-class classifiers comprises error-free normal data points. In contrast, the anomalies detected are labelled as the opposite class.

Recent studies have implemented one-class classifiers such as the *One-Class Support Vector Machine* (OC-SVM) [38,39] for sensor data anomaly detection, which maps the train data to a higher-dimensional feature space via a kernel function and tries to find a hyperplane with a maximum margin to encapsulate all of the normal train data. Lamrini et al. [40] proposed an anomaly detection method for network traffic characterisation using OC-SVM. Hejazi and Singh [41] compared one-class SVM with two-class SVM to show the effectiveness of the OC-SVM approach. For WSNs, Trinh et al. [42] used OC-SVM with the radial basis function kernel to detect anomalies. In 2021, Jia et al. [43] implemented OC-SVM on flight operation safety to detect anomalies and predict risk.

For local and unsupervised time series anomaly detection, a variation of PCA called the *One-Class Principal Component Classifier* (OC-PCA) was proposed in [21]. The approach is divided into two phases, with the first phase being the offline training phase, which trains a PCA model using normal data collected from each sensor, and the second phase being the online detection phase, where current observations are projected into the feature subspace and compared with a normal behaviour model based on a dissimilarity matrix. However, this approach excludes automated time series feature extraction and selection. Instead, these algorithms operate on the sensor measurements themselves. For datasets with a large amount of time series data, this becomes a problem as it becomes computationally expensive and causes overfitting of the PCA model.

*Local Outlier Factor* (LOF), proposed by Breunig et al. [31], is another state-of-the-art one-class detection method. LOF calculates the degree of anomaly of a sample based on the local densities of its neighbouring points [44]. Xu et al. [45] proposed a hierarchical framework using LOF to detect anomalies in WSNs. Ma et al. [46] used LOF on the PCA-projected domain for real-world, large-scale traffic data. Moreover, for computer networks, Auskalnis et al. [47] and Paulauskas and Bagdonas [48] implemented LOF to detect anomalies for intrusion detection application to detect cyberattacks.

Another one-class algorithm is *Isolation Forest* (IF), proposed by Liu et al. [49], inspired by Random Forest. It is based on binary trees which are constructed to isolate anomalies instead of learning the profiles of normal points. Susto et al. [50] applied an online IF for an industrial application, plasma etching. Furthermore, IF has been applied to detect anomalies in various applications such as gas turbines [51] and hydrological time series [52]. Cheng et al. [53] combined IF with LOF to detect anomalies and reduce time complexity.

Other anomaly detection methods are not based on one-class classifiers. Instead, they are clustering-based approaches such as *Density-Based Spatial Clustering of Applications with Noise* (DBSCAN). For clustering approaches to anomaly detection, studies [23,54,55] used DBSCAN to find unexpected patterns or behaviour of time series for temperature and multivariate weather data. In order to detect anomalies in electricity consumption behaviours, Zhang et al. [24] proposed DBSCAN with feature engineering. The feature engineering process also uses a feature extraction and selection method to extract a comprehensive set of features, and the feature selection is based on variance and the Maximum-Relevance Minimum-Redundancy technique with Maximal Information Coefficient.

DBSCAN is similar to LOF in that they both detect anomalies based on densities of the points and are completely unsupervised. However, DBSCAN only works on historical data and is not commonly applied to streaming data. It requires all of the data to be sent to the central server for anomaly detection, which might be too late depending on the IoT application. Ideally, erroneous data is not sent to the central server as it wastes the already limited bandwidth of the low-cost IoT sensor, and it is much more ideal to have quick detection of anomalies, as this could save ample time and resources.

Furthermore, there are supervised anomaly detection techniques such as ensemble classifiers [20], which use automated feature engineering to extract and select relevant features. Moreover, studies  [28,56] have proposed a Support Vector Classifier for real-time fault detection, which includes hand-picked features for feature engineering, which may not be generalisable across different sensor devices. However, these supervised methods require a labelled dataset in the training phase, making it difficult for anomaly detection in sensor data obtained from IoT applications such as environmental monitoring due to the lack of labelled datasets in this field.

A recent trend in anomaly detection involves *deep learning approaches*. Sinha and Das [29] incorporated modified deep reinforcement learning to detect and categorise different types of sensor errors, such as bias, drift, complete failure, and precision degradation. Pota et al. [57], Li et al. [58], and Goyal et al. [59] all proposed an autoencoder approach to detect anomalies in real time for different industrial and agricultural IoT applications. Furthermore, Liu et al. [60] presented a Convolutional Neural Network-based model to detect anomalies in industrial IoT applications accurately. However, deep learning methods are very data-intensive and some also require labelled data.

To address the gaps in knowledge, we propose AAAD, a machine learning framework that learns a sensor-specific normality model. AAAD builds on top of an unsupervised time series feature engineering approach [30], where the output of the feature engineering is used as input for a local outlier model.

## 3. Methodology

### 3.1. Background

Anomalies in IoT sensor data can happen in different forms, affecting sensor data quality. Teh et al. [32] provide a comprehensive description of the types of errors related to the term *anomalies* used in this paper. Defining normal behaviour as the expected high-quality data state, an anomaly is an observation that largely deviates from normality or is inconsistent with the rest of the dataset [21,33], which also includes faults that occur in sensor data, such as outliers, constant values, missing data, and drifts. These faults should be detected in order to improve sensor data quality. From a broader perspective, anomalies can be generally classified into three different categories: point anomaly, contextual anomaly, and collective anomaly [61,62]:

- A point anomaly occurs when an individual data point is considered anomalous when viewed against the entire dataset.
- A contextual anomaly is a data point that is anomalous in a specific context but not otherwise. An example of a contextual anomaly is a sensor time series with yearly temperature measurements where a temperature reading of 3 °C is not unusual for winter months but is a contextual anomaly if it occurs in the summer.
- A collective anomaly occurs when a collection of related data instances is anomalous regarding the entire dataset, where the individual data points might not be anomalies, but their occurrence together as a collection is anomalous.

**Figure 1:**
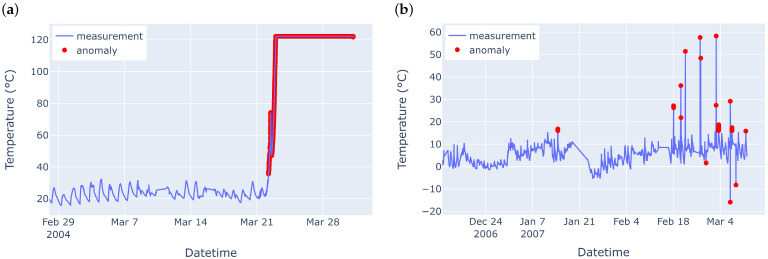
Examples of the temperature time series plots of (**a**) IBRL [63] Sensor 27 and (**b**) LUCE [64] Sensor 28, where the red dots represent the point anomalies (outliers and spikes).

Figure 1 shows an example of point anomalies (denoted by red dots) where (a) shows that outlier anomalies have very high temperature readings compared to the rest of the time series and (b) shows outliers in the form of spikes, having significantly different temperature readings from their neighbouring points and with respect to the whole time series.

**Figure 2:**
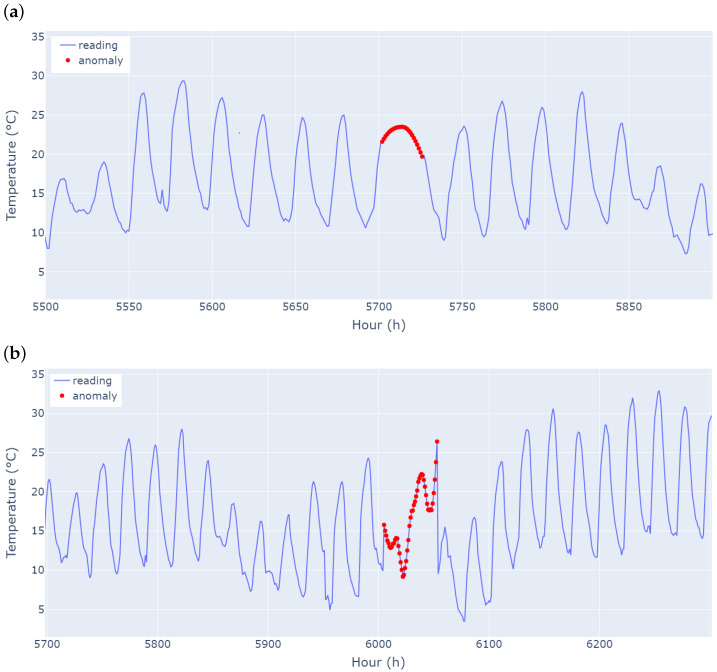
Contextual and collective anomalies in UCR (CIMIS) [65,66] dataset. (**a**) The merged peak anomaly is a contextual anomaly. (**b**) The smoothed random walk with anomalous peak is a collective anomaly.

An example of a contextual anomaly is seen in Figure 2a, where a real-world temperature reading is injected with an artificial anomaly where the two high-temperature peaks between hours 5702 and 5726 are joined. Figure 2b shows the real-world temperature data of a random two-day period between hours 6005 and 6053, where a collective anomaly is added. A section of the data is synthetically replaced with a smoothed random walk, making the daily patterns less noticeable, and an anomalous peak temperature is imputed at dawn. However, it is a period when the temperature is the coolest.

### 3.2. Sensor-Specific Normality Model

The sensor-specific normality model for anomaly detection is learnt from a calibration period of consecutive sensor readings, which is assumed to be anomaly-free. Before we start discussing the details on how to fit a sensor-specific normality model, we want to give an overview of a fitted normality model, which comprises three sub-models:

- The feature extraction vector function $\vec{v}:R^{T}\to R^{m}=F$, which is a tuple of *m* time series feature extraction functions $\vec{v}=\vec{v}\left(\vec{z}\right)=\left(v_{1}\left(\vec{z}\right),\ldots ,v_{m}\left(\vec{z}\right)\right)=\vec{x}$ characterising a sequence of *T* consecutive sensor readings $\vec{z}\in R^{T}$ by an *m*-dimensional feature vector $\vec{x}=\left(x_{1},\ldots ,x_{m}\right)\in R^{m}$.
- The standardisation model $\theta :R^{m}\to R^{m}$, with parameters $\vec{\mu }=\left(\mu_{1},\ldots ,\mu_{m}\right)$ and standard deviations $\vec{\sigma }=\left(\sigma_{1},\ldots ,\sigma_{m}\right)$, which characterises the expected means $\vec{\mu }$ and standard deviations $\vec{\sigma }$ along the axes of time series feature space F.
- The Local Outlier Factor model $\xi :R^{m}\to R$, returning the anomaly score $\xi (\bar{x})$ of standardised time series feature vector $\bar{x}=\left(\frac{x_{1}-\mu_{1}}{\sigma_{1}},\ldots ,\frac{x_{m}-\mu_{m}}{\sigma_{m}}\right)$.

After fitting the sensor-specific model normality model, the anomaly score $\zeta ({\vec{z}}_{t})$ of sensor readings ${\vec{z}}_{t}=\left(z_{t-T+1},\ldots ,z_{t}\right)$ can be computed as

$$\zeta \left({\vec{z}}_{t}\right)=\xi \left(\frac{v_{1}\left({\vec{z}}_{t}\right)-\mu_{1}}{\sigma_{1}},\ldots ,\frac{v_{m}\left({\vec{z}}_{t}\right)-\mu_{m}}{\sigma_{m}}\right).$$

**Figure 3:**
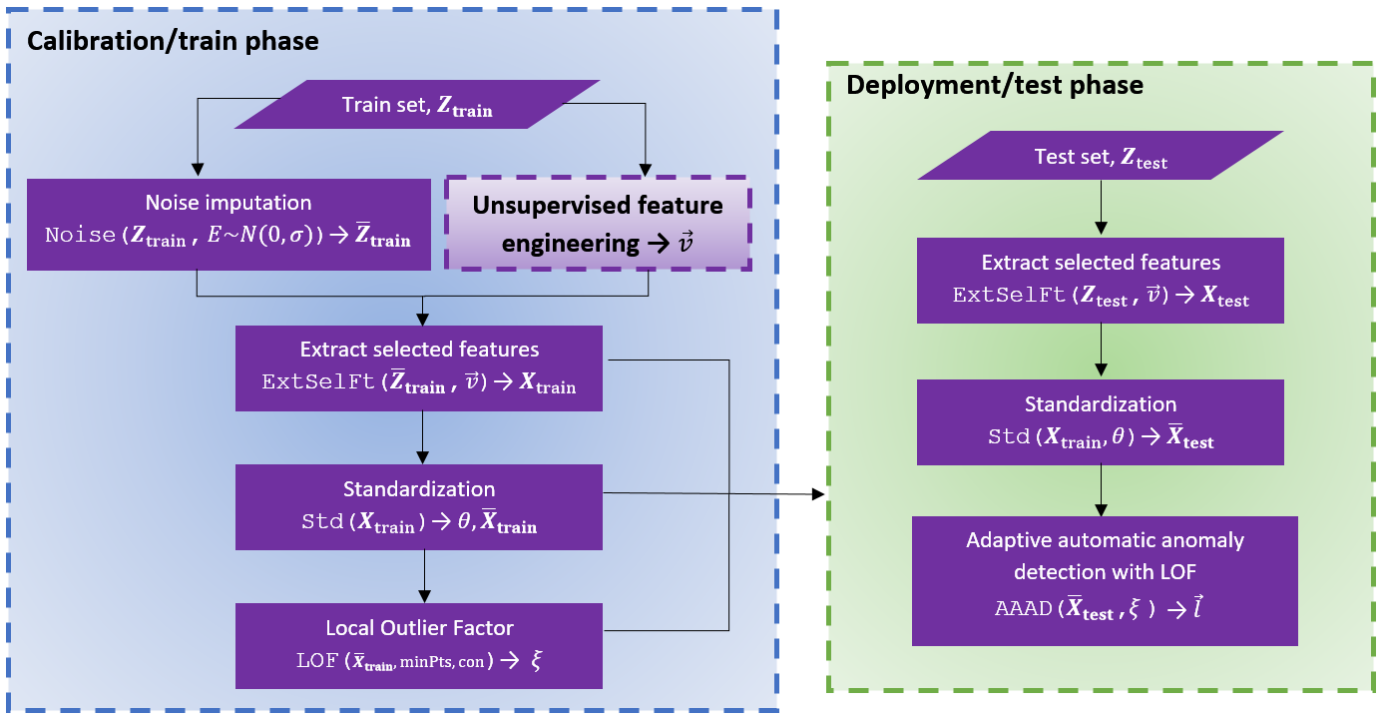
AAAD framework for fitting and deploying normality models. Feature matrix $Z_{\mathrm{train}}$ contains the raw sensor readings after decomposing the time series data of the calibration phase by a rolling window approach. Matrix $Z_{\mathrm{train}}$ is used for unsupervised feature engineering [30], determining the time series feature extraction vector function $\vec{v}$. In addition, randomly selected rows of $Z_{\mathrm{train}}$ are perturbed by Gaussian noise sampled from a normal distribution N(0,σ=1). Using $\vec{v}$, matrix ${\bar{Z}}_{\mathrm{train}}$ is converted into a time series feature matrix $X_{\mathrm{train}}$ from which the standardisation model θ is fitted. The standardised time series feature matrix ${\bar{X}}_{\mathrm{train}}$ is used to fit the Local Outlier Factor model ξ, which can be deployed together with $\vec{v}$ and θ. During deployment or testing, the raw sensor readings are decomposed into matrix $Z_{\mathrm{test}}$, converted into time series feature matrix $X_{\mathrm{test}}$ by $\vec{v}$, normalised to ${\bar{X}}_{\mathrm{test}}$ with θ, and row-wise processed with ξ. Vector $\vec{l}$ contains the anomaly scores of the test windows, also known as rows of $Z_{\mathrm{test}}$.

Fitting a sensor-specific normality model starts with recording the sensor readings during calibration. Using a rolling window decomposition, the sensor readings of the calibration period are arranged into a naïve feature matrix $Z_{\mathrm{train}}\in R^{N\times T}$ of *N* time series samples $\vec{z}\in R^{T}$. Following the unsupervised feature engineering approach introduced in [30], feature matrix $Z_{\mathrm{train}}$ is used to configure the feature extraction vector function $\vec{v}$ (Figure 3). The components of $\vec{v}$ are automatically selected from a set of 794 predefined mathematical functions, which have been found to generate good predictors in the context of applied time series machine learning [67]. The unsupervised feature selection process simulates a regression problem predicting statistics like mean or standard deviation of near-future values (NFV) from a given time series window ([30], p. 18038). The learning algorithm combines univariate and multivariate feature selection to choose an optimal subset of time series feature extraction functions for the given regression task [68]. Consequently, the configuration of the time series feature extraction vector function $\vec{v}$ depends on the configured regression problem, such that two different time series feature vector functions $v_{\mathrm{mean}}$ and $v_{\mathrm{std}}$ are considered in the experimental section.

AAAD extends the robustness of the unsupervised feature selection introduced in [30] by imputing some rows of $Z_{\mathrm{train}}$ with Gaussian noise E∼N(0,1) sampled from a normal distribution N(0,1) with a mean of zero and a standard deviation of one. Note that the noise amplitude is small compared to the scale of the sensor readings in the considered use cases (Figure 1 and Figure 2). The rows of $Z_{\mathrm{train}}$, which are perturbed by Gaussian noise, are determined by a Bernoulli process with probability p=0.1%, meaning that there is a 0.1% chance that Gaussian noise is added to a specific row of $Z_{\mathrm{train}}$ and a 99.9% chance that no noise is added.

The perturbed matrix ${\bar{Z}}_{\mathrm{train}}$ is converted into time series feature matrix $X_{\mathrm{train}}\in R^{N\times m}$ by applying feature extraction $\vec{v}$ row-wise. Every row of $X_{\mathrm{train}}$ is an *m*-dimensional time series feature vector $\vec{x}\in R^{m}$ characterising a specific time series window. Every column of $X_{\mathrm{train}}$ is a specific time series feature with *N* samples, which has been generated by applying a specific mathematical function to all *N* time series windows. The standardisation model θ is learned from $X_{\mathrm{train}}$ by computing the means $\vec{\mu }=\left(\mu_{1},\ldots ,\mu_{m}\right)$ and standard deviations $\vec{\sigma }=\left(\sigma_{1},\ldots ,\sigma_{m}\right)$ of the *m* time series feature columns of $X_{\mathrm{train}}$. These parameters are used to standardise the feature matrix $X_{\mathrm{train}}$ by subtracting the respective time series feature mean from every column of $X_{\mathrm{train}}$ and dividing the difference by the standard deviation of the respective feature. The resulting standardised matrix is named ${\bar{X}}_{\mathrm{train}}$.

The standardised selected features set ${\bar{X}}_{\mathrm{train}}$ is used as input to train a one-class classifier. Here, we use Local Outlier Factor (LOF) and denote the fitted model ξ (Section 3.3). The LOF is an adaptive anomaly detection model that independently determines the non-linear threshold to detect anomalies. This boundary in standardised feature space is the normality model, which separates normal and anomalous data for a specific sensor. In other words, in the calibration phase, AAAD can define the boundary between normal and anomalous data by learning the distances and densities of the normal anomaly-free train data, which should encapsulate all of the normal data points.

Once the calibration phase is completed, anomaly detection is performed in the deployment phase, where any new and unseen data from the test set is screened. In this phase, feature values are extracted from the test data based on the sensor-specific feature extraction $\vec{v}$, which was configured in the calibration phase. The matrix $X_{\mathrm{test}}$ of selected feature values of the test set is then standardised according to the standardisation model θ.

Finally, anomaly detection is performed on the standardised features set ${\bar{X}}_{\mathrm{test}}$ via the trained one-class LOF classifier ξ fitted in the calibration phase. The trained LOF model ξ has learnt an adaptive hypersphere in feature space enclosing all normal observations. New observations outside the hypersphere are considered an anomaly. It makes the automatic anomaly detection adaptive as the boundaries learnt are specific to an individual sensor’s normal behaviour. Moreover, the model’s non-linearity allows for a higher resolution detection than a linear anomaly detection model.

### 3.3. Local Outlier Factor

Local Outlier Factor (LOF) [31] is a one-class classifier used in the proposed AAAD framework. It is used to independently determine the boundary between the normal train data from the calibration phase and the normal or anomalous test data from the deployment phase. The one-class classifier requires only anomaly-free training data containing only one (normal) class and does not require any ground-truth labels, making it unsupervised.

LOF works by computing the degree of outlierness of a data point (in this case, the learned time series feature representation of a time series chunk) compared to its local neighbours. In LOF models, the locality is given by *k*-nearest neighbours, where the distances between the *k*-nearest neighbours are used to calculate the local density of the neighbourhood. The local deviation of a data point with respect to its neighbours is calculated, and if it deviates much further than the local density of its neighbours, it is considered an outlier.

Compared to other unsupervised anomaly detection methods, such as clustering, which focuses on finding clusters, LOF optimises outlier detection. Even for the DBSCAN clustering method, the notion of outliers is still a fixed binary, and there is no quantification of how outlying a data point is. Hence, LOF is robust in detecting anomalies as it can quantify the degree of irregularity of a data point. In contrast, the PCA used in [30] cannot find an adaptive threshold specific to each sensor device.

## 4. Datasets and Experiments

In order to evaluate the proposed sensor anomaly detection approach, three real-world publicly available datasets are used. The datasets are: Intel Berkeley Research Lab (IBRL) [63], Lausanne Urban Canopy Experiment (LUCE) [64], and the UCR Anomaly Benchmark Datasets 2021 [65,66], (UCR) [64], and UCR Anomaly Benchmark Datasets 2021 [65,66] (UCR). Only the LUCE dataset from the SensorScope project is used for evaluation, as it offers a wide array of sensor time series and has a more extended deployment period compared to the other datasets from SensorScope. The IBRL and LUCE are very similar in measuring environmental variables and have mostly point anomalies. However, the key difference is that IBRL is an indoor environment monitoring dataset, whereas SensorScope focuses on outdoor environment monitoring. The different geographical distribution of the environmental sensors gives a variety of sensor time series data available for evaluation, which might contain different types of anomalies. However, they do not have ground-truth labels indicating the anomalous readings; thus, they are labelled via semi-automatic heuristic labelling.

The UCR dataset, on the other hand, is a challenging dataset for time series anomaly detection, including the more complex collective and contextual anomalies. The authors carefully modelled and designed anomalies for a large set of real-world and synthetic time series to become a reliable benchmark for time series anomaly detection [69]. Time series in the UCR dataset have two types of anomalies inserted at random locations in the time series. Those anomalies are usually within the normal range of values with respect to the entire dataset, making it more challenging to detect such anomalies, for example, by using simple heuristics.

### 4.1. Experimental Set Up

The experiments were run on a single computer with a CPU of 2.2 GHz and 16 GB memory, and the experiment was planned and conducted following closely the steps introduced by Géron [70]. The Python version and versions of its respective libraries used are as follows:

- Python = 3.7.4.
- Pandas = 1.3.5.
- Numpy = 1.21.1.
- Plotly = 5.11.0.
- Scikit-learn = 0.23.2.
- Tsfresh = 0.16.0.

The AAAD framework with unsupervised feature engineering and adaptive thresholding with LOF is evaluated and compared against other state-of-the-art anomaly detection approaches. These approaches include clustering and one-class classifiers:

- One-cCass Principal Component Classifier (OC-PCA) [30], which finds the top two principal components and a linear threshold to separate the normal and anomalous data;
- Density-Based Spatial Clustering of Applications with Noise (DBSCAN) [23,24,54,55], which is a density-based clustering method that finds clusters of arbitrary shape and takes noise into account;
- One-Class Support Vector Machine (OC-SVM) [40,41,42,43], which projects the samples into a higher-dimensional space to find a hyperplane that separates normal and anomalous values;
- Isolation Forest (IF) [50,51,52,53] which recursively partitions or isolates a sample where the paths are shorter for anomalies when partitioned, as they are more isolated.

The models are evaluated using six different metrics, namely, False Positive Rate (FPR), Recall, Precision, F-score, Accuracy, and Matthews Correlation Coefficient (MCC) [32].

### 4.2. Dataset Preparation

#### 4.2.1. IBRL and LUCE

For the two indoor and outdoor monitoring datasets, IBRL and LUCE, semi-automatic labelling heuristics were used to label the time series for model evaluation. To emphasise, these labels are not used as part of the AAAD framework or in other classifiers. The heuristics are just a baseline measure of how well the classifiers perform compared to each other.

The minority labelling approach is used where a sample $x_{n}$ in a time series window ${\vec{z}}_{c}$ is flagged as an anomaly by the heuristics, then the entire chunk ${\vec{z}}_{c}$ will be considered an anomaly. The heuristics are as follows, where $x_{n}$ is labelled as an anomaly if it satisfies either one of two heuristics, which define the two anomalies: an outlier and a spike [32]:

1. The data point $x_{n}$ is more than three standard deviations away from the mean of the time series ${\vec{s}}_{m}$,
  (1) $$x_{n}\leq \mu_{m}-3\sigma_{m},\;x_{n}\geq \mu_{m}+3\sigma_{m}.$$
2. The difference between $x_{n}$ and its neighbouring readings is larger than the standard deviation of the time series ${\vec{s}}_{m}$,
  (2) $$|x_{n}-x_{n-1}|>\sigma_{m},\;\left|x_{n+1}-x_{n}\right|>\sigma_{m}.$$

The constant value anomaly heuristic is not included as it is trivial to determine constant value anomalies. Other than that, a pre-processing step is added where a time series chunk ${\vec{z}}_{c}$ with a gap of 5 consecutive minutes of missing values such that n=10 for a K=120 chunk (sampling rate of 30 s) or n=60 for a K=720 chunk (sampling rate of 5 s) are discarded. The pre-processing step cleans the data and removes chunks with missing data.

Every sensor device in IBRL and LUCE has its own sampling period, where the sensor devices in IBRL measure every 30 s, whereas the sampling period of sensor devices in LUCE is either 5 or 30 s. Given that it is a real-world dataset with underlying connectivity issues that cause data to arrive later than expected, the timeliness of the time series is inconsistent. Therefore, it is not guaranteed that a segmented window or chunk would have K=120 samples for a sensor with a 30 s sampling period or K=720 samples for a 5 s sampling period sensor.

It is a problem when the dimensions of the chunks are not consistent, especially for PCA, which is used for visualisation. To ensure a fair ground for comparison, each chunk is upsampled or downsampled depending on the number of samples in the chunk. Any chunk with less than K=120 samples (for a 30 s sampling period) or K=720 samples (for a 5 s sampling period) is upsampled by adding new samples using linear interpolation. Chunks of more than K=120 or K=720 samples are downsampled by removing samples with the smallest time difference between the neighbouring readings.

In summary, since only sensor devices with at least one anomaly in the time series are taken into account, and with the new pre-processing and heuristics, a total of M=49 sensor devices from IBRL and M=9 sensor devices from LUCE are considered in this experiment. The time series from IBRL consists of a one-month period (from 28 February 2004 to 5 April 2004), whereas the sensor time series from LUCE consists of readings over four months (from 1 December 2006 to 31 March 2007).

#### 4.2.2. UCR

In the UCR time series anomaly detection dataset, there is only one anomaly per time series, where the anomalies are simulated according to real-world errors. Although there is one anomaly per time series, the anomaly occurs in a section where the entire collection of data points in that section is considered anomalous. It is different from the single-point anomaly seen in IBRL and LUCE. Hence, because of the range nature of the anomalies, the scoring function is such that if any part of the anomaly is predicted as an anomaly (preferably the centre), it is considered correctly detected.

It is also safe to assume that the training data is free of anomalies and the test data has only one (range) anomaly. This study considers only the California Irrigation Management Information System (CIMIS) temperature dataset from UCR. It consists of a single time series of public weather data from the CIMIS Station 44 in Riverside, California. The CIMIS time series is chosen to ensure the consistency of the type of environment variable, as the AAAD was evaluated on the temperature readings for the other two environmental monitoring datasets, IBRL and LUCE. The CIMIS air temperature time series consists of hourly temperature readings for about ten years, from 2009 to 2019. There are five types of artificially imputed anomalies in the single UCR CIMIS temperature time series. Table 1 shows the chunk index of the anomalies according to the different types of anomalies artificially imputed.

The AAAD framework is evaluated on the UCR (CIMIS) dataset to detect more sophisticated anomalies. For this dataset, the chunk size of K=24 is selected, where each chunk contains 24 samples. Since the sampling period is one hour, each chunk corresponds to one day’s worth of temperature readings, where the daily pattern is expected to be learnt by the anomaly detection model. The same minority labelling approach is taken, such that if a chunk contains an anomalous sample, then the entire chunk is considered an anomaly. As long as one of the anomaly chunks is detected as an anomaly by the anomaly detection model, it is considered a valid detection.

The sliding window shift is also selected as Δ=6, i.e., six hours corresponding to a quarter of a day for a sampling period of one hour. The training data size is also set in accordance with the suggested portion, which is the first 4000 samples [66]. Since each chunk has a length of K=24, the training set consists of a=166 chunks, i.e., 3984 (slightly less than the recommended 4000) samples, whereas the remaining samples are for the test set. It is about half of the entire UCR CIMIS temperature time series.

The dataset is segmented into chunks of length K=24, and the train and test data are split accordingly. For the calibration or train phase, the AAAD framework described in Section 3 is applied. From the unsupervised feature engineering of the AAAD framework, a set of F=5 selected features is obtained, each for the two different target statistics, ${\vec{v}}_{\mathrm{mean}}$ and ${\vec{v}}_{\mathrm{std}}$. Only the sensor-specific case of AAAD is considered, as the CIMIS dataset only consists of a single sensor time series, thus rendering the deployment-specific case inapplicable. Furthermore, noise is not imputed in this dataset as the model has already seen much training data.

### 4.3. Hyperparameter Optimisation

Each of the classifiers has different sets of hyperparameters to tune, except OC-PCA, where the best threshold α is already found through a series of experiments in [30], which is α=13 and α=48 for IBRL and LUCE datasets, respectively. In order to ensure a fair comparison between all methods, a Leave-One-Sensor-Out-Cross-Validation or short Leave-One-Out Cross-Validation (LOOCV) is performed on the sensor time series for each dataset to determine the optimal hyperparameters for the other models and subsequently the best performance of a classifier. LOOCV is chosen as LUCE only has M=9 sensors left in the dataset after the dataset preparation. It returns *M* folds of different train and test sensor combinations. For each fold, the train sensors contain every sensor but one from the dataset, and the test sensor will be one left out. The test sensor is not used to train the model, and it is only used for evaluation.

#### 4.3.1. Local Outlier Factor (LOF)

The minPts hyperparameter, also known as n_neighbors or *k*, is the number of neighbours defining the neighbouring points. The range of values starts from 10, as the lower bound defined in the original paper by Breunig et al. [31], whereas the upper bound is the number of samples in the train set. The con hyperparameter is the amount of contamination of the train set, which indicates the proportion of outliers or noise in the train set. The `auto’ value for con is the default value in scikit-learn, the threshold defined in the original paper [31]. The lower bound is 0 (not inclusive), and the upper bound is 0.5. The range of values tested is in logarithmic increments, covering most values without being too computationally expensive. The best hyperparameters found are k=35 and con=0.001.

#### 4.3.2. Density-Based Spatial Clustering of Applications with Noise (DBSCAN)

According to the original paper by Sander et al. [71], the minPts or the number of neighbours should be set to twice the dimension of the dataset, which in our case is 20 (twice of F=10 selected features). The ϵ hyperparameter indicates how close the points should be to be considered as a cluster. Therefore, the optimal value can be found by applying the k-nearest neighbours algorithm to find the minPts-nearest neighbour distances for each point. The point of maximum curvature, also known as the knee or elbow of the sorted minPts-distance graph, is the optimal value of ϵ.

#### 4.3.3. One-Class Principal Component Classifier (OC-PCA)

With the kernel set to radial basis function to obtain a non-linear boundary for detecting anomalies, OC-SVM has two hyperparameters to tune, where γ is the kernel coefficient and ν is the upper bound of the fraction of training errors, similar to the con hyperparameter in LOF, as well as the lower bound of the fraction of support vectors. The ‘auto’ and ‘scale’ values for γ are the default values defined by scikit-learn where $scale=\frac{1}{\left(F\times X.\mathrm{var}\right)}$ and $auto=\frac{1}{F}$. The other values are in $log_{10}$ scale. Similarly, for ν, the lower bound is 0 (not inclusive) and the upper bound is 1 (inclusive). Therefore, all values from $10^{-10}$ to $10^{-1}$ on a logarithmic scale and values from 0.2 to 1 in a 0.1-step are tried and tested. The best hyperparameters found are $\gamma =10^{-10}$ and ν=0.05.

#### 4.3.4. Isolation Forest (IF)

There are three hyperparameters for IF models: the number *e* of base estimators in the model, the number *s* of samples to train each base estimator, and con, which are the amounts of contamination in the dataset. For this, the original paper by Liu et al. [49] found that the number of estimators converges well before 100; therefore, the range of values to be tested is from 10 to 100, with a step of 10. As a binary tree, the number of samples to draw for each tree has a $log_{2}$ scale increment, starting with the lower bound of 2 to the maximum number of samples in the train set. Like LOF, con is set from $10^{-10}$ to 0.5 on a logarithmic scale. The best hyperparameters are e=60, s=72, c=0.001.

## 5. Results and Discussion

The AAAD framework with unsupervised feature engineering and LOF classifier is compared with the framework proposed in [30] with linear static threshold, also known as OC-PCA, along with other state-of-the-art approaches for sensor data anomaly detection, which include clustering (DBSCAN) and one-class classifiers (OC-SVM and IF). The input for all classifiers is the small set of meaningful features selected via the unsupervised feature engineering technique [30] using the standard deviation of the adjacent window as the target value or, in short, ${\vec{v}}_{\mathrm{std}}$. The selected features ${\vec{v}}_{\mathrm{std}}$ are shown to be highly efficient and robust in learning the normal behaviour of a time series for both IBRL and LUCE datasets, being better than using raw data (naïve feature engineering).

In this experiment, different performance metrics were used in addition to MCC, including FPR, Recall, Precision, Accuracy, and F-score. All six performance metrics are described and their pros and cons discussed at length in [32]. The results of the experiment for IBRL, LUCE, and UCR are tabulated in Table 2 and will be discussed in more detail in the following subsections.

### 5.1. Performance Measure for Anomaly Detection

Measuring the performance of anomaly detection algorithms requires considering that anomaly detection problems are inherently imbalanced, because the ratio of anomalous samples to normal samples is typically small. The problem is that some performance measures might indicate a high performance, although the algorithm always predicts the majority class and classifies the samples predominantly as normal. In order to demonstrate the problem and discuss the performance of anomaly detection algorithms from the perspective of different applications, we briefly review six established classification performance measures before discussing the results of the experiments in detail. A more comprehensive overview in the context of sensor data quality can be found in [32] (p. 41f).

In general, the performance of anomaly detection algorithms needs to be measured out-of-sample on time series data, which have not been seen during the training process. Thus, a trained anomaly detection algorithm is presented with *N* different time series samples or windows and predicts for every sample whether the respective sample is an anomaly or represents normal data. Here, we are only considering binary predictions using the following symbols:

**True positives** TP is the number of time series samples correctly identified as anomalies.
**True negatives** TN is the number of time series samples correctly identified as normal.
**False positives** FP is the number of time series samples that are normal but incorrectly labelled as anomalies (type 1 error).
**False negatives** FN is the number of anomalous time series samples that were incorrectly identified as normal (type 2 error).

From this definition follows N=TP+TN+FP+FN. Established performance measures are as follows:

**False Positive Rate FPR** $=\frac{FP}{TN+FP}$, also known as fall-out or the false alarm rate, is useful for applications that focus on avoiding the misclassification of normal data as anomalous. A smaller FPR is better.
**Recall** $=\frac{TP}{TP+FN}$, also known as true-positive rate, sensitivity, or hit rate, is useful for applications that require that all anomalies are detected. At the same time, costs for classifying normal data as anomalous can be ignored. A larger Recall is better.
**Precision** $=\frac{TP}{TP+FP}$, also known as the positive predictive value, is useful for applications that associate high costs with false positives. A large Precision is better.
**F-Score** $=2\times \frac{\mathrm{Precision}\times \mathrm{Recall}}{\mathrm{Precision}+\mathrm{Recall}}$ is a harmonic mean of recall and precision. A larger F-score is better.
**Accuracy** $=\frac{TP+TN}{TP+TN+FP+FN}$ is very inaccurate for imbalanced problems, because predicting the majority class always results in high Accuracy (cf last row of Table 2).
**Matthews Correlation Coefficient MCC** $=\frac{TP\times TN-FP\times FN}{\sqrt{\left(TP+FP)(TP+FN)(TN+FP)(TN+FN\right)}}$ is well-suited to measure the performance of imbalanced classification problems [72]. Larger MCC scores are better. An MCC score of zero indicates guessing of the majority class.

The results are tabulated in Table 2, where the same six performance metrics are used to quantify the performance of the models, which are FPR, Recall, Precision, F-score, Accuracy, and MCC. It is evident that the AAAD method is the best-performing model across all six performance measures. It also scored a very high MCC at 0.840, significantly higher than the second-best performing model, OC-SVM. The FPR achieved is also 0%, which means there are no false positives, i.e., no normal data mislabelled as anomalies. Since there are no false positives, the Precision is also at 100%, meaning all anomalies were correctly detected over the total anomalies identified.

### 5.2. Point Anomalies in IBRL and LUCE

The experiment results on the IBRL datasets show that AAAD performs the best in almost all performance measures compared to the other anomaly detection methods. All performance scores are obtained after averaging the scores from each test fold of the Leave-One-Out Cross-Validation. It resulted in 347,472, 557,280, and 1,737,168 models built for AAAD, OC-SVM, and IF, respectively, where the hyperparameters are tuned according to the train folds. The final tuned model is then evaluated on the test fold, based on the best hyperparameters selected using the corresponding train fold. Since the other two methods, OC-PCA and DBSCAN, work on the entire dataset where the hyperparameters can be easily found, it is performed on each sensor device without the LOOCV, and the results are extracted.

Here, MCC is focused on as it is more robust for imbalanced datasets that take into account all four cells of the confusion matrix [72]. AAAD has a very high MCC value of 0.968, better than OC-PCA, which has an MCC of 0.931. In addition, OC-PCA determines the threshold retrospectively after all data has been seen, where it finds a linear and static threshold. Comparatively, AAAD is more sophisticated as it adaptively determines a non-linear threshold for each sensor device.

The AAAD framework also outperforms the other one-class classifiers, OC-SVM and IF, which did not score well in MCC. AAAD has a slight FPR, meaning it has some false alarms, i.e., normal points that are incorrectly predicted as anomalies. However, it is still relatively low, at a rate of 0.767%. OC-PCA and DBSCAN each have an FPR of 0.3%, whereas IF has the highest FPR of 14%. The bad performance is also reflected in its MCC score. OC-SVM has 0% FP; however, it lacks in terms of MCC performance compared to AAAD.

The experimental results show that taking only a part of the confusion matrix into account can be misleading, such as Accuracy, where almost all classifiers show excellent performance. However, hiding behind the scenes is an imbalanced dataset, with one class (normal) dominating the other (anomaly), which is even more prominent in LUCE. Predicting all points as normal (TN) can also result in high Accuracy. MCC or F-score is much more reliable, giving an actual representation of the performance, though F-score does not consider true negatives, which may also be vulnerable.

To reinforce the results from the IBRL dataset, the same experiment is run on the LUCE dataset, with results obtained from the test folds via LOOCV. In total, 11,097, 17,793, and 55,449 models were created to tune the hyperparameters and evaluate AAAD, OC-SVM, and IF results, respectively. It can be seen once again that AAAD has the best MCC and is consistently very good across all five other metrics. It also outperforms OC-PCA, indicating that different sensor devices have different normal sensor behaviours, which requires adaptively choosing the threshold for individual sensor devices.

**Figure 4:**
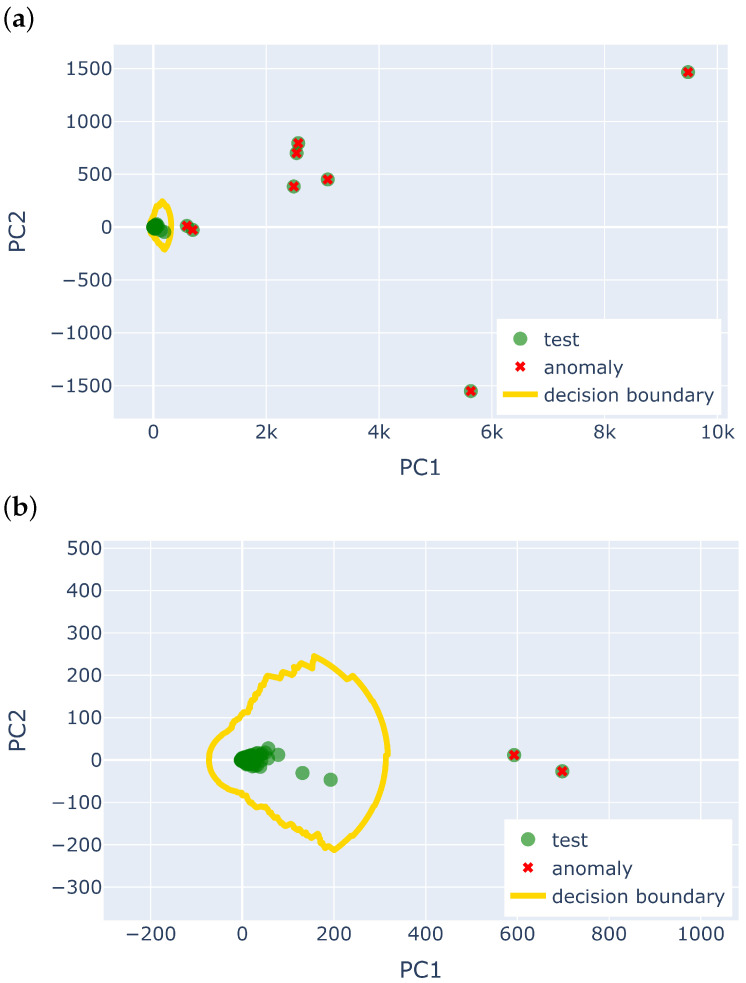
Normality model of AAAD for Sensor 29 of the LUCE dataset. (**a**) Decision boundary of LOF projected onto the first two principal components of the extracted time series features. Data points (green dots) outside the boundary are classified as anomalies (red crosses). (**b**) Zoomed-in view highlighting the non-linear nature of the decision boundary.

It also suggests that a non-linear boundary is better for the LUCE dataset, where the temperature is more variable due to the volatility of measurements from sensors placed outdoors. Figure 4 shows the non-linear decision boundary learnt by the LOF model in the AAAD framework, visualised in two-dimensional PCA space. Note that in the actual AAAD framework, LOF is trained on the *F*-dimensional selected feature space from ${\vec{v}}_{\mathrm{std}}$, and PCA is just used for visualisation.

Another interesting point is that the Accuracy of AAAD is slightly below that of a few of the other anomaly detection methods, such as DBSCAN. However, as explained previously, Accuracy is not a robust performance measure, and LUCE is an extreme case of class imbalance. Some sensor time series in LUCE only have one anomaly out of thousands of data points, making the class imbalance more prominent. It is further demonstrated in other metrics, where the other anomaly detection methods have a low F-score, MCC, and high FPR.

Though AAAD and DBSCAN have a similar performance in terms of MCC, the significant difference is that AAAD has only seen three days’ worth of training data. In contrast, DBSCAN requires the entire dataset to find clusters. The small train set is a significant advantage of the proposed anomaly detection method compared to DBSCAN, and it can potentially be deployed to detect anomalies online without requiring prior knowledge of the entire dataset.

The AAAD also has the highest Recall, similar to IF, meaning that more predicted anomalies are correctly predicted. Furthermore, AAAD also has the highest Precision, meaning that more actual anomalies are predicted correctly. Although AAAD scored a slightly higher FPR in the LUCE dataset than the IBRL dataset, once again demonstrating the complexity of the LUCE dataset, the F-score is the highest among all other methods. The good performance scores once again prove that AAAD, though having seen only a small section of the time series, can outperform other clustering and one-class classifier solutions for sensor data anomaly detection.

**Figure 5:**
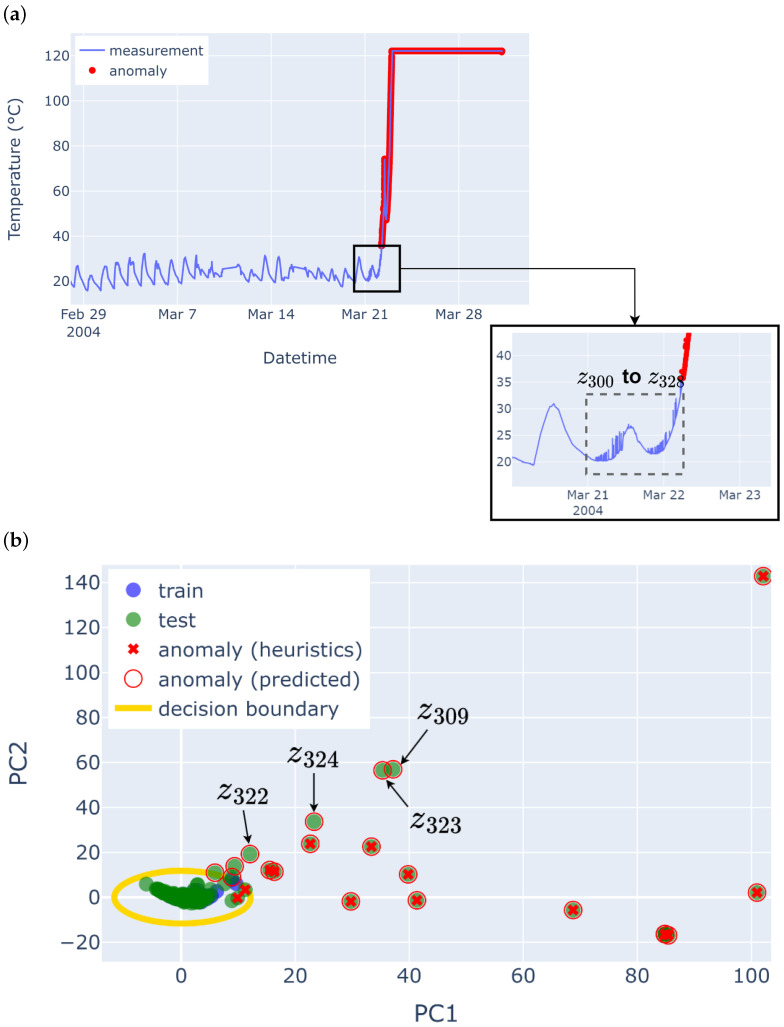
IBRL Sensor 27—temperature time series and PCA plot. (**a**) The time series of IBRL Sensor 27, zooming in to 21 May–22 May, corresponds to chunks $z_{300}$ to $z_{328}$. (**b**) The PCA subspace of IBRL Sensor 27 shows the training data (blue dots), the test data (green dots), the heuristic anomaly labels (red cross), the predicted anomaly labels (red circle), and the LOF decision boundary (yellow curve). Note that most training data (blue dots) are hidden behind the test data (green dots). Some green test data points, which are labelled as normal by the heuristics, are located outside the decision boundary. Those correspond to chunks $z_{309}$, $z_{322}$, $z_{323}$, and $z_{324}$.

However, the experiments conducted on the IBRL and LUCE datasets are subject to some risk of bias due to the use of semi-automatic labelling via heuristics. The AAAD approach has not shown its full potential due to the limitation in the heuristics used for labelling (Section 4.2), which might not be able to capture all anomalies as it focuses on point anomalies. An example of this is seen in Figure 5a, which is the time series plot of IBRL Sensor 27 with the anomalies labelled via the semi-automatic heuristics used in the evaluation. Zooming in to a section of the time series, it is seen that chunks $z_{300}$ to $z_{328}$ contain some noise anomaly (little fluctuations in the time series), occurring around 21 March to 22 March, indicating the start of the sensor failure.

The noise anomaly can be categorised as a contextual anomaly, as the readings are within the normal range of temperature values but are anomalous in this specific context. However, the heuristics, which focus on outlier-based point anomaly labelling, could not capture such local contextual anomaly in the time series. With that being said, the AAAD approach was able to pick up these anomalies. Figure 5b shows the visualisation of the PCA plot obtained from the selected features of the AAAD. The blue points are the training data used to train the model, which helps define the LOF decision boundary, in which anything within the yellow line is considered normal data. It is seen that there are a few green test data points that are further away from the centre of the normal cluster in the PCA subspace and are outside the decision boundary. These green data points with a red circle correspond to chunks $z_{309}$, $z_{322}$, $z_{323}$, and $z_{324}$, which are predicted as anomalies by AAAD, showing that the selected features were able to detect the irregularity seen in Figure 5a where the heuristics could not.

The anomaly labelling via the heuristics approach used for evaluation is primarily a baseline to measure how well the AAAD and other anomaly detection methods perform compared to each other and provide a foundation for the comparison. On top of that, AAAD can detect anomalies not captured by the heuristics, proving that anomaly detection is not as simple as defining heuristics. Therefore, AAAD is also evaluated on the UCR dataset, which covers these types of more complex anomalies, to fully evaluate the robustness of the proposed framework.

In summary, the AAAD method performs very well and is robust across all evaluation metrics for both IBRL and LUCE datasets. It also solves the novelty detection problem by learning a normality model. It represents the expected dynamics or normal behaviour pattern of a sensor time series by learning from anomaly-free, normal training data. In the proposed approach, the training set is small compared to the test set. Once the anomaly detection model is trained, any new and unseen data can be potentially scanned online and detected as an anomaly if it has a different pattern from the learned normal behaviour model. The AAAD method can also define the anomaly threshold independently across different sensor devices in a non-linear fashion, enabling individual sensors to be assigned individualised decision boundaries automatically without human intervention.

### 5.3. Contextual and Collective Anomalies in UCR

It is seen that the AAAD model with ${\vec{v}}_{\mathrm{mean}}$ unsupervised feature engineering method works well for almost all anomaly types, except for (iii) noise, where the features selected were not able to distinguish the anomaly from normal data. The features from ${\vec{v}}_{\mathrm{std}}$, on the other hand, do not work as well as there is sometimes a false positive at chunk $z_{192}$. However, it could detect the noise anomaly, unlike its ${\vec{v}}_{\mathrm{mean}}$ counterpart. Since both versions contain meaningful features that help detect different types of anomalies, a merge between the two target statistics, ${\vec{v}}_{\mathrm{mean}}+\;+\;{\vec{v}}_{\mathrm{std}}$, is performed, where the features selected by the two target statistics are taken into account.

**Figure 6:**
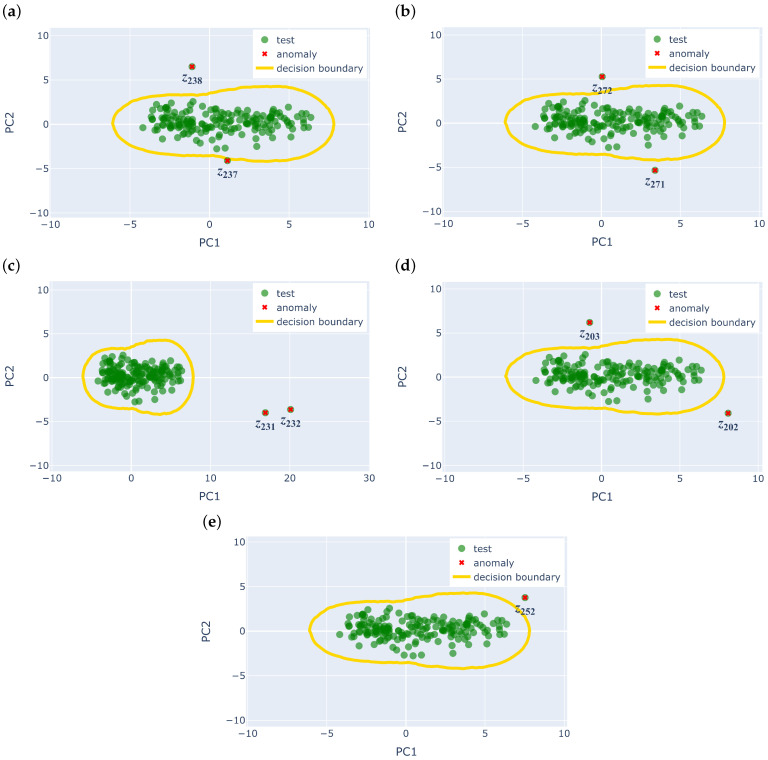
UCR (CIMIS)—PCA plot of ${\vec{v}}_{\mathrm{mean}}+\;+\;{\vec{v}}_{\mathrm{std}}$. By merging the selected features, AAAD with ${\vec{v}}_{\mathrm{mean}}+\;+\;{\vec{v}}_{\mathrm{std}}$ can successfully detect all five types of anomalies. The indices of the respective anomaly chunks are given in Table 1. (**a**) Merge peak and remove valley. (**b**) Flipped data across mean. (**c**) Noise. (**d**) Random walk. (**e**) Smoothed random walk and anomalous peak.

Table 3 and Table 4 show the selected features of ${\vec{v}}_{\mathrm{mean}}$ and ${\vec{v}}_{\mathrm{std}}$, respectively. There are nine features instead of ten for ${\vec{v}}_{\mathrm{mean}}+\;+\;{\vec{v}}_{\mathrm{std}}$, as ${\vec{v}}_{\mathrm{mean}}$ and ${\vec{v}}_{\mathrm{std}}$ both have maximum as one of their features, which is a duplicate. The combination of the two target statistics is shown to have found a better separation between normal values and anomalies, as seen in Figure 6, where all five types of anomalies are successfully detected, and there are no false positives. PCA is only used here for visualisation, and the yellow curve shows the threshold found by the LOF model, where points outside this decision boundary are labelled as anomalies. It indicates that the target statistics is an important parameter that needs to be carefully selected depending on the application. However, the mean and standard deviation of the rolling time series window work well in all three datasets (IBRL, LUCE, and UCR).

In order to evaluate the performance of AAAD, the proposed framework is compared with other state-of-the-art anomaly detection methods, similar to the previous experiments for IBRL and LUCE. The four other methods used for comparison are OC-PCA, DBSCAN, OC-SVM, and IF. The hyperparameters used for all methods except for OC-PCA and DBSCAN are based on the optimal hyperparameters used for the LUCE dataset, which is also an outdoor monitoring dataset. The optimal hyperparameter for the OC-PCA method is α=6.5, which was obtained via hyperparameter sweeping the entire dataset.

In summary, AAAD is evaluated on an additional dataset, UCR, to study the effectiveness of the proposed approach in detecting different types of anomalies. These anomalies are more complex as they are usually within the range of normal values of the time series. They differ from the point anomalies seen in the two previous datasets, IBRL and LUCE, where the anomalies have values significantly different from the rest of the dataset. The UCR dataset is a real-world dataset with simulated anomalies and actual ground-truth labels, which is created as a benchmark to allow meaningful comparisons.

The anomalies generated in this dataset are more sophisticated as they are primarily contextual and collective anomalies, with the anomalies being within the normal range of values. From evaluating the AAAD framework with unsupervised feature engineering and LOF on this dataset, it is seen that AAAD can robustly detect other types of complex anomalies. It also performs significantly better than other state-of-the-art anomaly detection methods such as DBSCAN, OC-SVM, and IF. Once different types of anomalies can be accurately detected, many opportunities for improving sensor data quality are opened up. Future research could look into categorising and differentiating the types of anomalies detected, which would give further insight into the detected anomalies.

## 6. Conclusions and Future Work

After reading this article on training and evaluating normality models for environmental IoT sensors, the reader might wonder: What is normal? What is normality? Moreover, how do humans evaluate normality? Research in cognitive science suggests that humans learn prescriptive and descriptive norms and integrate them into an internal representation of their normality [73]. This observation might explain why human experts can easily spot anomalous sensor signals. They have learned what a normal sensor signal looks like. In other words, human experts have learnt a model of normality, which can be effectively used to spot anomalous sensor signals.

For artificial agents, the task of recognising anomalies in sensor signals is much more complex and mainly relies on having at least some anomaly examples such that supervised or semi-supervised machine learning techniques can perform function approximation to discriminate normal and anomalous sensor signals [74,75]. While normality models have started to gain traction in the context of deep learning [76], these learning algorithms still require either labelled data or large training sets to fit the model.

In this work, we introduce a very different approach, which assumes that anomaly-free sensor readings have been recorded over a short period (e.g., three days) directly after the installation of the sensor. This condition is easily fulfilled in most practical applications because sensors are typically installed manually, and the respective engineer would conduct a post-commissioning check confirming the sensor measurements’ normality by simply inspecting the recordings. Based on a small calibration dataset, the AAAD framework learns a normality model of the sensor readings, which can reliably detect anomalous signals without having observed any anomalies before. The AAAD framework learns the sensor-specific normality model from unsupervised time series feature engineering in combination with a descriptive model (standardisation) and a prescriptive model (one-class classifier). We tested the capability of AAAD on three public datasets of environmental sensor data (IBRL, LUCE, UCR). We compared its performance on anomaly detection with four other established anomaly detection algorithms (OC-PCA, DBSCAN, OC-SVM, IF). The experiments show that AAAD outperforms the other algorithms regarding Recall, F-score, and MCC, which strongly indicates that AAAD will become a new standard for anomaly detection in IoT applications. Another benefit of AAAD is that the sensor-specific time series feature space is interpretable, because the underlying mathematical functions are all well-defined and can provide further insights to domain experts and expand the field of explainable artificial intelligence.

Further analysis will involve a sensitivity analysis of AAAD concerning hyperparameters like the probability of the Bernoulli process and the amplitude and standard deviation of imputed noise. Up to this point, AAAD has only been evaluated on environmental sensor data, indicating that the range of applications might be restricted to systems exhibiting a natural cycle of readings. Applications of AAAD for anomaly detection in industrial and health settings will be sought to map its generalisability. The algorithmic simplicity of the learned normality model indicates that there is the potential for the calibrated normality model to be deployed onto an edge device and tested for streaming data. Therefore, the next steps in this research will involve systematic analysis of the spatial and temporal complexity of the AAAD framework both in the calibration and deployment phases, which will also improve our understanding of the opportunities for the deployment to edge devices, online learning, and mitigating concept drift, e.g., due to seasonal effects. Another possible research direction is given by multivariate data, opening the applications to anomaly detection in more complex systems. The final goal will be to publish AAAD as an open-source machine learning library, which will be available to the community.

The perspective of learning individualised normality models opens new opportunities for anomaly detection applications in signal processing, and we are optimistic that AAAD will become the foundation for a new generation of anomaly detection applications.

## Figures and Tables

**Table 1:** UCR (CIMIS)—five types of synthetic anomalies. The types of synthetic anomalies found in the UCR (CIMIS) dataset [65,66] and the index of the anomalous chunks after the time series is segmented into one-day chunks. Types (i) and (v) are visualised in Figure 2a and Figure 2b, respectively.

Number	Anomaly Type	Anomaly Chunk Index
(i)	Merge peaks and remove valley	237–238
(ii)	Flipped data across mean	271–272
(iii)	Noise	231–233
(iv)	Random walk	202–204
(v)	Smoothed random walk and anomalous peak	250–252

**Table 2:** Anomaly detection performance. Performance of AAAD anomaly detection framework against OC-PCA and other recent solutions for anomaly detection, DBSCAN, OC-SVM, and IF for IBRL, LUCE, and UCR datasets. Bold values indicate the best performing algorithms. The AAAD framework has the best F1-score (between 5.4% and 9.3% better than the follow-up) and the best MCC score (between 4.0% and 7.6% better than the follow-up).

Dataset	Features	Classifier	FPR	Recall	Precision	F-Score	Accuracy	MCC
IBRL	${\vec{v}}_{\mathrm{std}}$	AAAD	0.007	**0.991**	0.999	**0.995**	**0.991**	**0.968**
		OC-PCA	0.003	0.948	0.937	0.944	0.962	0.931
		DBSCAN	0.003	0.805	0.839	0.811	0.980	0.810
		OC-SVM	**0.0**	0.942	**1.0**	0.959	0.949	0.886
		IF	0.142	0.866	0.975	0.915	0.865	0.599
LUCE	${\vec{v}}_{\mathrm{std}}$	AAAD	0.051	**0.958**	**0.998**	**0.977**	0.959	**0.802**
		OC-PCA	0.004	0.656	0.628	0.646	0.986	0.662
		DBSCAN	**0.0003**	0.781	0.827	0.730	**0.993**	0.762
		OC-SVM	0.045	0.761	0.776	0.768	0.773	0.643
		IF	0.356	**0.959**	0.979	0.968	0.941	0.508
UCR	${\vec{v}}_{\mathrm{std}}+\;+\;{\vec{v}}_{\mathrm{mean}}$	AAAD	**0.0**	**0.733**	**1.0**	**0.82**	**0.995**	**0.840**
		OC-PCA	0.001	0.466	0.9	0.593	0.990	0.632
		DBSCAN	0.005	**0.733**	0.633	0.666	0.989	0.669
		OC-SVM	**0.0**	0.633	**1.0**	0.75	0.994	0.781
		IF	0.169	0.799	0.064	0.119	0.829	0.193

**Table 3:** Features selected using $FS_{mean}$ (UCR). The five time series features selected using the proposed sensor-specific unsupervised feature selection approach using $FS_{mean}$ as a target statistic for detecting anomalies in the UCR dataset.

Feature	tsfresh Algorithm	Parameters
Maximum	maximum	None
Quantile	quantile	q=0.6
Quantile	quantile	q=0.7
Quantile	quantile	q=0.8
Conditional Dynamics	change_quantiles	f_agg="mean", isabs=False,
		qh=1.0, ql=0.0

**Table 4:** Features selected using $FS_{std}$ (UCR). The five time series features selected using the proposed sensor-specific unsupervised feature selection approach using $FS_{std}$ as a target statistic for detecting anomalies in the UCR dataset.

Feature	tsfresh Algorithm	Parameters
Maximum	maximum	None
Complexity	cid_ce	normalize=False
Conditional Dynamics	change_quantiles	f_agg="var", isabs=False,
		qh=0.6, ql=0.0
Conditional Dynamics	change_quantiles	f_agg="var", isabs=False,
		qh=0.8, ql=0.0
Conditional Dynamics	change_quantiles	f_agg="var", isabs=True,
		qh=1.0, ql=0.0

## Data Availability

Data derived from public domain resources.
